# 5′-Amino-1,3-dioxo-2′,3′-di­hydro-7′*H*-spiro­[indane-2,7′-thieno[3,2-*b*]pyran]-6′-carbonitrile 1′,1′-dioxide

**DOI:** 10.1107/S1600536810010019

**Published:** 2010-03-24

**Authors:** Shun-Hua Wang, Yue-Ning Jiang, Jiang-Na Zhang

**Affiliations:** a School of Mechatronic Engineering, Lanzhou Jiaotong University, Lanzhou 730070, People’s Republic of China; b School of Civil Engineering, Lanzhou Jiaotong University, Lanzhou 730070, People’s Republic of China

## Abstract

The title compound, C_16_H_10_N_2_O_5_S, was synthesized *via* the condesation of dihydro­thio­phen-3(2*H*)-one 1,1-dioxide, 1*H*-indene-1,2,3-trione and malononitrile in ethanol. The 2,3-dihydro­thio­phene 1,1-dioxide and pyran rings adopt envelope conformations. The mean planes through the planar part of the pyran ring and the benzene ring are nearly perpendicular, forming a dihedral angle of 88.40 (7)°. The crystal packing is stabilized by inter­molecular N—H⋯O and N—H⋯N hydrogen bonds with the sulfone O atom and the cyano N atom acting as acceptors.

## Related literature

For the uses of thienopyranyl compounds such as thieno[3,2-*b*]pyran derivatives as anti­viral agents and α-2C adreno­receptor agonists, see: Chao *et al.* (2009 ▶); Friary *et al.* (1991 ▶). For puckering parameters, see: Cremer & Pople (1975 ▶).
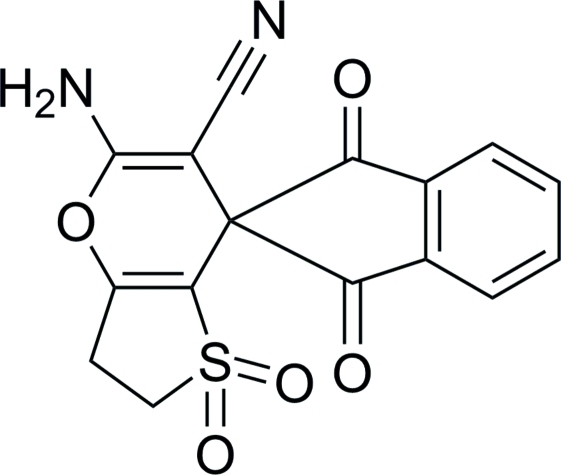


## Experimental

### 

#### Crystal data

C_16_H_10_N_2_O_5_S
*M* *_r_* = 342.32
Monoclinic, 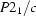

*a* = 9.436 (3) Å
*b* = 10.602 (3) Å
*c* = 14.777 (4) Å
β = 99.137 (4)°
*V* = 1459.6 (6) Å^3^
*Z* = 4
Mo *K*α radiation
μ = 0.25 mm^−1^
*T* = 116 K
0.28 × 0.20 × 0.18 mm

#### Data collection

Rigaku Saturn CCD area-detector diffractometer
Absorption correction: multi-scan (*CrystalClear*; Rigaku/MSC, 2002 ▶) *T*_min_ = 0.933, *T*_max_ = 0.956
9619 measured reflections
2549 independent reflections
1985 reflections with *I* > 2σ(*I*)
*R*_int_ = 0.068

#### Refinement

*R*[*F* ^2^ > 2σ(*F* ^2^)] = 0.058
*wR*(*F* ^2^) = 0.127
*S* = 1.00
2549 reflections
226 parameters
3 restraints
H atoms treated by a mixture of independent and constrained refinement
Δρ_max_ = 0.58 e Å^−3^
Δρ_min_ = −0.64 e Å^−3^

### 

Data collection: *CrystalClear* (Rigaku/MSC, 2002 ▶); cell refinement: *CrystalClear*; data reduction: *CrystalClear*; program(s) used to solve structure: *SHELXS97* (Sheldrick, 2008 ▶); program(s) used to refine structure: *SHELXL97* (Sheldrick, 2008 ▶); molecular graphics: *SHELXTL* (Sheldrick, 2008 ▶); software used to prepare material for publication: *SHELXTL*.

## Supplementary Material

Crystal structure: contains datablocks I, global. DOI: 10.1107/S1600536810010019/hg2656sup1.cif

Structure factors: contains datablocks I. DOI: 10.1107/S1600536810010019/hg2656Isup2.hkl

Additional supplementary materials:  crystallographic information; 3D view; checkCIF report

## Figures and Tables

**Table 1:** Hydrogen-bond geometry (Å, °)

*D*—H⋯*A*	*D*—H	H⋯*A*	*D*⋯*A*	*D*—H⋯*A*
N1—H1*C*⋯N2^i^	0.89 (1)	2.23 (1)	3.067 (3)	157 (2)
N1—H1*D*⋯O2^ii^	0.89 (1)	2.03 (1)	2.865 (2)	156 (2)

Symmetry codes: (i) 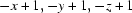; (ii) 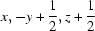.

**Fig. 1.**
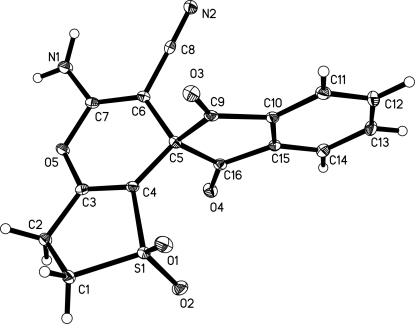
The structure of (I), showing 30% probability displacement ellipsoids and the atom-numbering scheme.

**Fig. 2.**
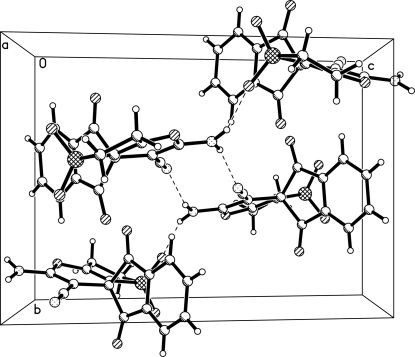
The packing diagram of (I). Intermolecular hydrogen bonds are shown as dashed lines.

## supplementary crystallographic information

### Comment

Thienopyranyl compounds, such as thieno [3,2-b]pyran derivatives, can be used as antiviral agents (Friary *et al.*, 1991) and α-2 C adrenoreceptor agonists ( Chao *et al.*, 2009). This led us to pay attention to the synthesis and bioactivity of these compounds. During the synthesis of thieno[3,2-b]pyran derivatives, the title compound, (I) was isolated and its structure was determined by X-ray diffraction. Here we report its crystal structure.

The molecular structure of (I) is shown in Fig. 1. In the molecular structure, the thiophene ring is in envelope conformation, for the deviation of C1 from the C2/C3/C4/S1 plane is 0.364 (3)Å with r.m.s. of 0.0056.

The pyran ring adopts an envelope conformation with atome C5 deviating from the C3/C4/C6/C7/O5 plane 0.228 (3) Å. According to Cremer & Pople analysis (Cremer & Pople, 1975), the puckering amplitude (Q) is 0.168 (2) Å. Its θ and φ are 103.2 (7) and 349.6 (7)°, respectively. The weighted planes of the pyran and phenyl rings are nearly perpendicular, with the dihedral angle between them 88.40 (7)°.  The five membered ring of 1*H*-indene-1,3(2*H*)-dione fragment adopts an envelope conformation, for the deviation of C5 from the C9/C10/C15/C16 plane is 0.149 (3)Å with r.m.s. of 0.0033. The crystal packing is stabilized by intermolecular hydrogen bonds: N1—H1C···N2, N1—H1D···O2(Fig.2 & Table 1).

### Experimental

The title compound was synthesized by the reaction of dihydrothiophen-3(2*H*)-one-1,1-dioxide (1 mmol), 1*H*-indene-1,2,3-trione (1 mmol) and malononitrile (1 mmol) in 10 ml ethanol under reluxing until completion (monitored by TLC). Cooling the reaction mixture slowly gave single crystals suitable for X-ray diffraction.

### Refinement

The hydrogen atoms bonded to the nitrogen atom were positioned from a Fourier difference map. The N—H bond lengths were restrained to 0.90Å with an estimated standard deviation 0.01. The distance between H1C and H1D was restrained to 1.50Å with an estimated standard deviation 0.01. Other H atoms were placed in calculated positions, with C—H = 0.93 or 0.97 Å, and included in the final cycles of refinement using a riding model, with U_iso_(H) = 1.2U_eq_(parent atom).

### Crystal data

**Table:**

C_16_H_10_N_2_O_5_S	*F*(000) = 704
*M**_r_* = 342.32	*D*_x_ = 1.558 Mg m^−^^3^
Monoclinic, *P*2_1_/*c*	Mo *K*α radiation, λ = 0.71073 Å
Hall symbol: -P 2ybc	Cell parameters from 5046 reflections
*a* = 9.436 (3) Å	θ = 1.4–27.9°
*b* = 10.602 (3) Å	µ = 0.25 mm^−^^1^
*c* = 14.777 (4) Å	*T* = 116 K
β = 99.137 (4)°	Prism, colorless
*V* = 1459.6 (6) Å^3^	0.28 × 0.20 × 0.18 mm
*Z* = 4	

### Data collection

**Table:**

Rigaku Saturn CCD area-detector diffractometer	2549 independent reflections
Radiation source: rotating anode	1985 reflections with *I* > 2σ(*I*)
multilayer	*R*_int_ = 0.068
Detector resolution: 14.63 pixels mm^-1^	θ_max_ = 25.0°, θ_min_ = 2.2°
ω and φ scans	*h* = −11→11
Absorption correction: multi-scan (*CrystalClear*; Rigaku/MSC, 2002)	*k* = −12→12
*T*_min_ = 0.933, *T*_max_ = 0.956	*l* = −17→11
9619 measured reflections	

### Refinement

**Table:**

Refinement on *F*^2^	Secondary atom site location: difference Fourier map
Least-squares matrix: full	Hydrogen site location: inferred from neighbouring sites
*R*[*F*^2^ > 2σ(*F*^2^)] = 0.058	H atoms treated by a mixture of independent and constrained refinement
*wR*(*F*^2^) = 0.127	*w* = 1/[σ^2^(*F*_o_^2^) + (0.0872*P*)^2^] where *P* = (*F*_o_^2^ + 2*F*_c_^2^)/3
*S* = 1.00	(Δ/σ)_max_ = 0.001
2549 reflections	Δρ_max_ = 0.58 e Å^−^^3^
226 parameters	Δρ_min_ = −0.64 e Å^−^^3^
3 restraints	Extinction correction: *SHELXL97* (Sheldrick, 2008), Fc^*^=kFc[1+0.001xFc^2^λ^3^/sin(2θ)]^-1/4^
Primary atom site location: structure-invariant direct methods	Extinction coefficient: 0.287 (14)

### Special details

**Table:**

Geometry. All esds (except the esd in the dihedral angle between two l.s. planes) are estimated using the full covariance matrix. The cell esds are taken into account individually in the estimation of esds in distances, angles and torsion angles; correlations between esds in cell parameters are only used when they are defined by crystal symmetry. An approximate (isotropic) treatment of cell esds is used for estimating esds involving l.s. planes.
Refinement. Refinement of *F*^2^ against ALL reflections. The weighted *R*-factor wR and goodness of fit *S* are based on *F*^2^, conventional *R*-factors *R* are based on *F*, with *F* set to zero for negative *F*^2^. The threshold expression of *F*^2^ > σ(*F*^2^) is used only for calculating *R*-factors(gt) etc. and is not relevant to the choice of reflections for refinement. *R*-factors based on *F*^2^ are statistically about twice as large as those based on *F*, and *R*- factors based on ALL data will be even larger.

### Fractional atomic coordinates and isotropic or equivalent isotropic displacement parameters (Å^2^)

**Table:**

	*x*	*y*	*z*	*U*_iso_*/*U*_eq_	
S1	0.97876 (6)	0.43469 (5)	0.18224 (4)	0.0160 (2)	
O1	0.95541 (18)	0.55710 (13)	0.14090 (11)	0.0236 (4)	
O2	0.95173 (17)	0.32826 (14)	0.12109 (11)	0.0253 (4)	
O3	0.68230 (16)	0.65052 (13)	0.24233 (10)	0.0205 (4)	
O4	0.68085 (16)	0.20574 (13)	0.21541 (10)	0.0216 (4)	
O5	0.93203 (15)	0.34731 (13)	0.42896 (10)	0.0183 (4)	
N1	0.7756 (2)	0.36526 (18)	0.52595 (13)	0.0224 (5)	
N2	0.4414 (2)	0.46284 (18)	0.40031 (13)	0.0242 (5)	
C1	1.1509 (2)	0.42085 (19)	0.25136 (15)	0.0170 (5)	
H1A	1.2145	0.3705	0.2204	0.020*	
H1B	1.1936	0.5034	0.2644	0.020*	
C2	1.1251 (2)	0.35644 (19)	0.33972 (15)	0.0172 (5)	
H2A	1.1471	0.2672	0.3379	0.021*	
H2B	1.1854	0.3937	0.3922	0.021*	
C3	0.9718 (2)	0.37524 (18)	0.34620 (14)	0.0151 (5)	
C4	0.8818 (2)	0.41635 (18)	0.27348 (14)	0.0148 (5)	
C5	0.7227 (2)	0.42359 (18)	0.26815 (15)	0.0149 (5)	
C6	0.6933 (2)	0.41396 (18)	0.36651 (15)	0.0150 (5)	
C7	0.7939 (2)	0.37715 (18)	0.43869 (14)	0.0160 (5)	
C8	0.5539 (2)	0.43993 (18)	0.38449 (15)	0.0160 (5)	
C9	0.6480 (2)	0.54337 (19)	0.22236 (15)	0.0158 (5)	
C10	0.5243 (2)	0.50111 (19)	0.15446 (14)	0.0151 (5)	
C11	0.4185 (2)	0.5739 (2)	0.10321 (15)	0.0201 (5)	
H11	0.4207	0.6615	0.1074	0.024*	
C12	0.3101 (2)	0.5133 (2)	0.04602 (15)	0.0233 (6)	
H12	0.2375	0.5604	0.0118	0.028*	
C13	0.3079 (3)	0.3814 (2)	0.03878 (15)	0.0226 (5)	
H13	0.2340	0.3425	−0.0005	0.027*	
C14	0.4129 (2)	0.3084 (2)	0.08852 (14)	0.0197 (5)	
H14	0.4117	0.2210	0.0831	0.024*	
C15	0.5215 (2)	0.36997 (18)	0.14740 (14)	0.0152 (5)	
C16	0.6443 (2)	0.31490 (19)	0.20917 (14)	0.0156 (5)	
H1C	0.6992 (18)	0.394 (2)	0.5484 (15)	0.036 (7)*	
H1D	0.841 (2)	0.325 (2)	0.5651 (14)	0.047 (9)*	

### Atomic displacement parameters (Å^2^)

**Table:**

	*U*^11^	*U*^22^	*U*^33^	*U*^12^	*U*^13^	*U*^23^
S1	0.0134 (4)	0.0201 (4)	0.0148 (3)	−0.0004 (2)	0.0034 (2)	−0.0006 (2)
O1	0.0225 (10)	0.0262 (9)	0.0231 (9)	0.0053 (6)	0.0064 (7)	0.0088 (7)
O2	0.0227 (10)	0.0316 (9)	0.0226 (9)	−0.0070 (7)	0.0062 (7)	−0.0101 (7)
O3	0.0213 (10)	0.0148 (8)	0.0257 (9)	−0.0017 (6)	0.0046 (7)	−0.0003 (7)
O4	0.0219 (9)	0.0154 (8)	0.0268 (9)	0.0029 (6)	0.0016 (7)	0.0006 (7)
O5	0.0147 (9)	0.0241 (8)	0.0167 (8)	0.0048 (6)	0.0043 (6)	0.0037 (6)
N1	0.0222 (12)	0.0287 (11)	0.0171 (10)	0.0084 (9)	0.0056 (9)	0.0052 (9)
N2	0.0179 (12)	0.0353 (11)	0.0196 (11)	0.0024 (8)	0.0039 (9)	−0.0013 (8)
C1	0.0133 (12)	0.0185 (11)	0.0191 (12)	−0.0018 (9)	0.0029 (9)	−0.0009 (9)
C2	0.0152 (12)	0.0183 (10)	0.0181 (11)	0.0014 (9)	0.0025 (9)	0.0007 (9)
C3	0.0175 (12)	0.0131 (10)	0.0157 (11)	0.0008 (8)	0.0051 (9)	−0.0015 (9)
C4	0.0135 (12)	0.0162 (10)	0.0156 (11)	−0.0006 (8)	0.0053 (9)	−0.0018 (9)
C5	0.0124 (12)	0.0157 (10)	0.0167 (11)	−0.0001 (8)	0.0027 (9)	0.0002 (9)
C6	0.0141 (12)	0.0150 (10)	0.0167 (11)	0.0009 (8)	0.0047 (9)	0.0005 (9)
C7	0.0168 (12)	0.0127 (10)	0.0196 (12)	0.0011 (8)	0.0065 (9)	−0.0003 (9)
C8	0.0185 (13)	0.0163 (11)	0.0125 (11)	−0.0024 (9)	0.0005 (9)	0.0002 (8)
C9	0.0140 (12)	0.0193 (11)	0.0158 (11)	0.0014 (8)	0.0074 (9)	0.0002 (9)
C10	0.0139 (12)	0.0187 (11)	0.0136 (11)	0.0013 (9)	0.0050 (9)	0.0010 (9)
C11	0.0217 (13)	0.0201 (11)	0.0192 (12)	0.0054 (9)	0.0052 (10)	0.0012 (9)
C12	0.0215 (14)	0.0305 (12)	0.0168 (12)	0.0077 (10)	−0.0007 (10)	0.0042 (10)
C13	0.0189 (13)	0.0309 (12)	0.0169 (12)	−0.0023 (10)	−0.0008 (10)	−0.0034 (10)
C14	0.0214 (13)	0.0205 (11)	0.0175 (12)	−0.0008 (9)	0.0040 (10)	−0.0001 (9)
C15	0.0138 (12)	0.0187 (10)	0.0135 (11)	−0.0001 (8)	0.0038 (9)	−0.0001 (9)
C16	0.0143 (12)	0.0179 (11)	0.0158 (11)	−0.0012 (9)	0.0064 (9)	0.0017 (9)

### Geometric parameters (Å, °)

**Table:**

S1—O1	1.4364 (15)	C4—C5	1.493 (3)
S1—O2	1.4428 (16)	C5—C6	1.526 (3)
S1—C4	1.756 (2)	C5—C9	1.554 (3)
S1—C1	1.782 (2)	C5—C16	1.559 (3)
O3—C9	1.205 (2)	C6—C7	1.368 (3)
O4—C16	1.207 (2)	C6—C8	1.409 (3)
O5—C3	1.367 (2)	C9—C10	1.482 (3)
O5—C7	1.371 (3)	C10—C11	1.388 (3)
N1—C7	1.334 (3)	C10—C15	1.394 (3)
N1—H1C	0.893 (9)	C11—C12	1.378 (3)
N1—H1D	0.889 (9)	C11—H11	0.9300
N2—C8	1.149 (3)	C12—C13	1.403 (3)
C1—C2	1.527 (3)	C12—H12	0.9300
C1—H1A	0.9700	C13—C14	1.374 (3)
C1—H1B	0.9700	C13—H13	0.9300
C2—C3	1.478 (3)	C14—C15	1.396 (3)
C2—H2A	0.9700	C14—H14	0.9300
C2—H2B	0.9700	C15—C16	1.477 (3)
C3—C4	1.333 (3)		
			
O1—S1—O2	116.12 (10)	C9—C5—C16	102.61 (17)
O1—S1—C4	111.29 (10)	C7—C6—C8	117.51 (19)
O2—S1—C4	109.41 (9)	C7—C6—C5	123.58 (19)
O1—S1—C1	112.48 (10)	C8—C6—C5	118.87 (19)
O2—S1—C1	110.42 (10)	N1—C7—C6	126.9 (2)
C4—S1—C1	95.13 (10)	N1—C7—O5	110.57 (19)
C3—O5—C7	116.39 (16)	C6—C7—O5	122.56 (18)
C7—N1—H1C	124.3 (15)	N2—C8—C6	178.7 (2)
C7—N1—H1D	119.4 (16)	O3—C9—C10	127.1 (2)
H1C—N1—H1D	116.3 (15)	O3—C9—C5	125.3 (2)
C2—C1—S1	105.81 (14)	C10—C9—C5	107.55 (16)
C2—C1—H1A	110.6	C11—C10—C15	120.68 (19)
S1—C1—H1A	110.6	C11—C10—C9	128.46 (19)
C2—C1—H1B	110.6	C15—C10—C9	110.84 (17)
S1—C1—H1B	110.6	C12—C11—C10	118.3 (2)
H1A—C1—H1B	108.7	C12—C11—H11	120.8
C3—C2—C1	106.59 (17)	C10—C11—H11	120.8
C3—C2—H2A	110.4	C11—C12—C13	120.8 (2)
C1—C2—H2A	110.4	C11—C12—H12	119.6
C3—C2—H2B	110.4	C13—C12—H12	119.6
C1—C2—H2B	110.4	C14—C13—C12	121.3 (2)
H2A—C2—H2B	108.6	C14—C13—H13	119.3
C4—C3—O5	124.2 (2)	C12—C13—H13	119.3
C4—C3—C2	119.78 (19)	C13—C14—C15	117.7 (2)
O5—C3—C2	116.05 (18)	C13—C14—H14	121.1
C3—C4—C5	124.48 (19)	C15—C14—H14	121.1
C3—C4—S1	108.20 (16)	C10—C15—C14	121.09 (19)
C5—C4—S1	126.58 (16)	C10—C15—C16	110.13 (17)
C4—C5—C6	106.25 (17)	C14—C15—C16	128.78 (19)
C4—C5—C9	116.60 (17)	O4—C16—C15	127.8 (2)
C6—C5—C9	109.32 (16)	O4—C16—C5	124.15 (19)
C4—C5—C16	112.07 (16)	C15—C16—C5	108.03 (16)
C6—C5—C16	109.94 (16)		
			
O1—S1—C1—C2	135.04 (14)	C3—O5—C7—C6	10.1 (3)
O2—S1—C1—C2	−93.46 (15)	C7—C6—C8—N2	59 (11)
C4—S1—C1—C2	19.47 (15)	C5—C6—C8—N2	−123 (11)
S1—C1—C2—C3	−20.47 (19)	C4—C5—C9—O3	−51.7 (3)
C7—O5—C3—C4	−7.2 (3)	C6—C5—C9—O3	68.8 (3)
C7—O5—C3—C2	173.23 (16)	C16—C5—C9—O3	−174.58 (19)
C1—C2—C3—C4	13.2 (3)	C4—C5—C9—C10	131.25 (19)
C1—C2—C3—O5	−167.28 (16)	C6—C5—C9—C10	−108.26 (18)
O5—C3—C4—C5	−7.5 (3)	C16—C5—C9—C10	8.4 (2)
C2—C3—C4—C5	172.05 (18)	O3—C9—C10—C11	−3.5 (4)
O5—C3—C4—S1	−178.14 (15)	C5—C9—C10—C11	173.4 (2)
C2—C3—C4—S1	1.4 (2)	O3—C9—C10—C15	177.91 (19)
O1—S1—C4—C3	−129.26 (15)	C5—C9—C10—C15	−5.1 (2)
O2—S1—C4—C3	101.07 (16)	C15—C10—C11—C12	0.6 (3)
C1—S1—C4—C3	−12.70 (16)	C9—C10—C11—C12	−177.8 (2)
O1—S1—C4—C5	60.3 (2)	C10—C11—C12—C13	−0.9 (3)
O2—S1—C4—C5	−69.34 (19)	C11—C12—C13—C14	0.3 (3)
C1—S1—C4—C5	176.89 (18)	C12—C13—C14—C15	0.6 (3)
C3—C4—C5—C6	16.4 (3)	C11—C10—C15—C14	0.3 (3)
S1—C4—C5—C6	−174.71 (14)	C9—C10—C15—C14	178.97 (19)
C3—C4—C5—C9	138.5 (2)	C11—C10—C15—C16	−179.47 (19)
S1—C4—C5—C9	−52.6 (2)	C9—C10—C15—C16	−0.8 (2)
C3—C4—C5—C16	−103.7 (2)	C13—C14—C15—C10	−0.9 (3)
S1—C4—C5—C16	65.2 (2)	C13—C14—C15—C16	178.8 (2)
C4—C5—C6—C7	−13.2 (3)	C10—C15—C16—O4	−173.9 (2)
C9—C5—C6—C7	−139.8 (2)	C14—C15—C16—O4	6.3 (4)
C16—C5—C6—C7	108.3 (2)	C10—C15—C16—C5	6.4 (2)
C4—C5—C6—C8	169.09 (17)	C14—C15—C16—C5	−173.4 (2)
C9—C5—C6—C8	42.5 (2)	C4—C5—C16—O4	45.5 (3)
C16—C5—C6—C8	−69.4 (2)	C6—C5—C16—O4	−72.4 (2)
C8—C6—C7—N1	−1.5 (3)	C9—C5—C16—O4	171.39 (18)
C5—C6—C7—N1	−179.2 (2)	C4—C5—C16—C15	−134.76 (18)
C8—C6—C7—O5	178.80 (17)	C6—C5—C16—C15	107.32 (18)
C5—C6—C7—O5	1.0 (3)	C9—C5—C16—C15	−8.9 (2)
C3—O5—C7—N1	−169.63 (17)		

### Hydrogen-bond geometry (Å, °)

**Table:**

*D*—H···*A*	*D*—H	H···*A*	*D*···*A*	*D*—H···*A*
N1—H1C···N2^i^	0.89 (1)	2.23 (1)	3.067 (3)	157 (2)
N1—H1D···O2^ii^	0.89 (1)	2.03 (1)	2.865 (2)	156 (2)

Symmetry codes: (i) −*x*+1, −*y*+1, −*z*+1; (ii) *x*, −*y*+1/2, *z*+1/2.
